# Recent greening may curb urban warming in Latin American cities of better economic conditions

**DOI:** 10.1016/j.landurbplan.2023.104896

**Published:** 2023-12

**Authors:** Yang Ju, Iryna Dronova, Daniel A. Rodriguez, Maryia Bakhtsiyarava, Irene Farah

**Affiliations:** a School of Architecture and Urban Planning, Nanjing University, Rm. 810, Jianliang Bldg., No. 22 Hankou Rd., Nanjing, China; b Department of Environmental Science, Policy, and Management, University of California, Berkeley, USA; c Department of Landscape Architecture and Environmental Planning, University of California, Berkeley, USA; d Department of City and Regional Planning, University of California, Berkeley, USA; e Institute of Transportation Studies, University of California, Berkeley, USA; f Institute of Urban and Regional Development, University of California, Berkeley, USA

**Keywords:** Urban warming, Economic conditions, Green space, Mediating effect, Temporal variability, Latin America

## Abstract

Rising temperatures have profound impacts on the well-being of urban residents. However, factors explaining the temporal variability of urban thermal environment, or urban warming, remain insufficiently understood, especially in the Global South. Addressing this gap, we studied the relationship between city-level economic conditions and urban warming, and how urban green space mediated this relationship, focusing on 359 major Latin American cities between 2001 and 2022. While effect sizes varied by economic and temperature measures used, we found that better economic conditions were associated with lower baseline greenness in 2011, which contributed to faster warming. There was modest evidence that this faster warming associated with lower baseline greenness and improved economic conditions was partially offset by cooling from recent greening (2001–2022) in cities of better economic conditions. This offset was more evident in arid cities. Together, these findings provide insights into the urban warming mechanism manifested through the effect of economic conditions on urban green space, for Latin American cities and other high-density cities transforming in a similar context.

## Highlights

• Links among economic conditions, green space and warming were assessed for Latin American cities.
• Better economic conditions exacerbate warming via historical loss of green space.
• Greening in economically developed cities partially curbs warming.
• These patterns vary by economic indicator, temperature measures and city subgroups.

## 1 Introduction

Globally, urban areas are experiencing unprecedented warming or temperature increases over time (Myint et al., 2013, Zhao et al., 2014), creating major risks for human health and well-being (Gasparrini et al., 2017, Jenerette et al., 2016, Weber et al., 2015). Among other factors, increased energy use and emissions from urbanization and economic development are contributing to greater urban warming (Imhoff et al., 2010, Myint et al., 2013, Stewart and Oke, 2012). By providing cooling from shading and evaporation, urban green space has been increasingly used as a means to regulate urban thermal environment (Massaro et al., 2023). However, urban green space is not evenly distributed within and between cities; rather, studies have found socioeconomic disparities of urban green space among communities and cities (Casey et al., 2017, Ju et al., 2021). Despite some efforts (Jenerette et al., 2006; Y. Li et al., 2023, Yin et al., 2023), it remains insufficiently understood how these urban green space disparities further affect urban thermal environment. Addressing this research gap is of especially great importance for Latin American cities, which have high urbanization rates (over 80% population living in urban areas, United Nations et al., 2019), large social disparities (Quistberg et al., 2018), and elevated heat-related mortality risk in hot temperatures (Kephart et al., 2022). In this context, this study provides insights into the relationships among urban thermal environment, city-level economic conditions, and urban green space in Latin American cities. Though these efforts, urban policy and management will be able to identify the drivers of urban warming and to better target cities in the greatest need of urban greening programs.

The relationship between urban thermal environment, city-level economic conditions, and the availability of urban green space is complex and multi-faceted. Generally, advanced industrial cities are associated with greater anthropogenic heat releases and transformations of blue and green spaces into built-up areas. These may together raise urban temperature by several degrees relative to the surroundings, known as the urban heat island effect (J. Li et al., 2011; Y. Li et al., 2020, Stewart and Oke, 2012). However, these cities may be more resourceful in regulating their thermal environment through urban greening (e.g. adding green spaces and improving upon existing ones), energy efficiency programs, and increasing the reflectance of surfaces (Ju et al., 2021, Richards et al., 2017, Yang et al., 2014; X. Zhou & Wang, 2011).

The connection between urban greening and economic conditions may lead to disparities in the distribution of urban green space. These disparities have been studied at the neighborhood scale, with findings summarized as the “luxury effect” and “deprivation amplification” (Leong et al., 2018, Macintyre, 2007, Schell et al., 2020), with some recent research focusing on these disparities at the city scale (Ju et al., 2021; Y. Li et al., 2023). Given the well-documented cooling effect of green space (Ziter et al., 2019) and the potential associations between economic conditions and green space, it is possible that green space affects the relationship between city-level economic conditions and urban thermal environment in two simultaneous but opposite directions. The historical removal of green space in advanced industrial cities due to economic development and urban expansion leads to rising temperature. However, greening measures in these cities may curb the temperature rise. With some exceptions (Jenerette et al., 2006, Yin et al., 2023), these two pathways forming the mediating effect of urban green space on the association between economic conditions and urban thermal environment, is rarely studied.

Another understudied topic is the temporal variability of urban thermal environment and its drivers. Studies so far have focused on explaining the spatial variability of urban thermal environment with economic indicators and green space (Jesdale et al., 2013, Richards et al., 2017). However, the evidence on the temporal variability of urban thermal environment (urban warming hereafter) and its drivers is scarce for a large sample of cities of different climate zones and economic development levels. One obstacle towards such investigations is the lack of consistent longitudinal temperature data with large spatial coverage for the cities.

Spatially explicit datasets on air temperature from climate reanalysis (e.g. Muñoz-Sabater et al., 2021) and satellite-derived land surface temperature (LST) provide opportunities to obtain wall-to-wall, longitudinal temperature measures for large samples of cities (Clinton & Gong, 2013; D. Zhou et al., 2019). While air temperature and LST tend to correlate with each other, they differ in physical interpretation and spatiotemporal characteristics of their respective data products. Climate reanalysis provides indicators such as air temperature (Hersbach, 2019, Papangelis et al., 2012, Sharma et al., 2017). However, the spatial resolution of climate reanalysis to date is typically too coarse (i.e., often greater than 5 km) to capture fine-scale intra-urban climate variations, which are influenced by features such as green space, buildings, and land uses. Satellite-derived LST provides an alternative to retrieve finer-scale historical temperature (Bechtel et al., 2019, Clinton and Gong, 2013, Imhoff et al., 2010, Spronken-Smith and Oke, 1998, Voogt and Oke, 2003), although one limitation of satellite-derived LST is missing observations due to cloud cover. The compatibility between temperature trends measured from air temperature and LST may vary across urban geographies, depending on contributions of different warming agents (Oyler et al., 2016, Wang et al., 2017). Generally, this compatibility is expected to increase when urban warming directly stems from land cover changes detectable by satellites. This would be the case with reduction in green space to provide cooling and expansion of impervious surfaces promoting absorption of solar radiation during the day and heat releases at night (Imhoff et al., 2010, Stewart and Oke, 2012, Zhao et al., 2014). However, other heat sources, including those from vehicle emissions and economic activities, are loosely associated with land cover changes (Juruš et al., 2016) and therefore are harder to detect with satellite-based LSTs.

Importantly, urban LST and its interpretation also differ between daytime and nighttime (Nichol, 2005, Oke, 1976). Daytime LST is often more sensitive to contrasts between hotter urban surfaces and cooler photosynthetically active vegetation (Azevedo et al., 2016, Nichol, 2005, Oke, 1976, Wang et al., 2017, Ziter et al., 2019), and in some cases daytime LST reflects the snapshot artifacts of building and tree shadows (Wang et al., 2017). In contrast, nighttime LST is more representative of heat releases from the energy absorbed during the day and broader-scale advective processes at spatial scales beyond individual buildings and trees (Nichol, 2005, Oyler et al., 2016, Wang et al., 2017, Ziter et al., 2019). Together, these nuances between air temperature, daytime LST, and nighttime LST highlight the need for a comprehensive assessment of these temperature indicators while considering their unique sensitivities to environmental and economic drivers of urban warming.

**Fig. 1:**
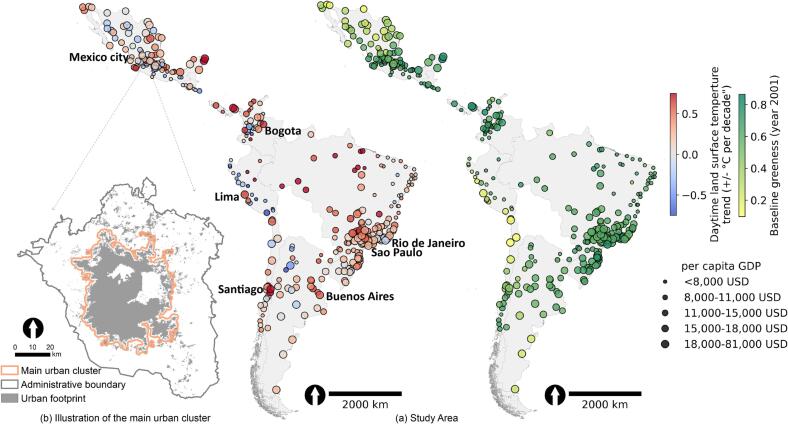
(a) Map of cities included and (b) illustration of the main urban cluster. In (a), colors represent the trend of daytime land surface temperature and baseline greenness (year 2001), and marker size represents per capita GDP.

To better understand how different factors shape urban thermal environment while considering the nuances between temperature measures, we investigate the associations between economic conditions and trends of air temperature and daytime and nighttime LSTs (i.e. urban warming), and the mediating effect of urban green space on these associations. Our sample consists of 359 geographically and socioeconomically diverse major Latin American cities from 10 countries (Fig. 1(a)). These cities constitute the study area of the Salud Urbana en América Latina (SALURBAL; Urban Health in Latin America) project (Quistberg et al., 2018), which investigates the socioeconomic and environmental determinants of urban health. Using path analysis in structural equation models, we addressed three research questions: (1) what is the relationship between city-level economic conditions and urban warming? (2) how does urban green space mediate this relationship? and (3) how do the findings from (1) and (2) vary by different economic development levels and climate zones?

## 2 Methods

### 2.1 Analysis units and timeframe

Instead of defining a city with its administrative boundary, we defined the city by its main urban cluster outlined by the SALURBAL study. The main urban cluster is the largest contiguous built-up area in a city (Fig. 1(b)). Therefore, we considered it as the hotspot of anthropogenic activities and consequently urban warming. Defining cities by main urban clusters is also in line with similar efforts across nations such as the EU-OECD delineation of functional urban area (Dijkstra et al., 2019). We collected the data and calculated each variable by the main urban cluster, when possible. However, economic indicators including per capita GDP and Social Environment Index (SEI) were only available by administrative boundaries (Table S1). Thus, in this article, we refer to “city” as its main cluster rather than its administrative boundary, unless we specify it differently. The timeframe in this study is from 2001 to 2022 as determined by the temporal availability of different datasets (Table S1). Since we did not have longitudinal data for some economic indicators of interest, we performed a cross-sectional analysis in this study. More details on how different variables are derived are provided in the sections below.

### 2.2 Outcome variable: temperature trend

For city-level temperature, we used three types of measures, including atmospheric temperature (T_air_) and daytime and nighttime LST (LST_day_ and LST_night_). Datasets for atmospheric temperature and LST are in gridded format, with each grid cell containing multiple observations over time and each city containing multiple grid cells. We summarized T_air_ as the area-mean (i.e. averaged over all grid cells in a city) of annual-averages (averaged across all observations over time in a grid cell) of daily average temperatures from the ERA5-Land reanalysis dataset (Copernicus Climate Change Service (C3S), 2019). We obtained LST data from MODIS terra land surface temperature 8-day product at 1 km resolution (MOD11A2.061). In a similar manner to T_air_, we calculated area-mean of annual average LST_day_ and LST_night_ for each city and year. The end-product here was city-specific time-series of T_air_, LST_day_, and LST_night_ between 2001 and 2022.

We then calculated the temperature trend as Sen’s slope of the city-specific time-series of T_air_, LST_day_, and LST_night_. Compared with slope from linear fit of the temperature time series, Sen’s slope is less sensitive to outliers and more robust to non-normal distributions (Sen, 1968). The city-specific temperature trend was used as the outcome in this study, with a positive trend indicating urban warming and a negative trend indicating urban cooling.

### 2.3 Exposure variables and mediators

**Economic indicators as exposures**. To answer the first research question on the associations between economic conditions and warming, we introduced four exposure variables to measure economic conditions. These variables were per capita Gross Domestic Product (GDP), total carbon footprint (Moran et al., 2018), a composite Social Environment Index (SEI) (Bilal et al., 2021), and nighttime light intensity (NTL).

First, per capita GDP was obtained from a global dataset developed by Kummu et al. (2018). It should be noted that the Kummu et al. dataset estimates per capita GDP by 1st-order administrative units (e.g. provinces Argentina and states in Brazil). Therefore, cities in the same 1st-order administrative unit share the same per capita GDP value (Gennaioli et al., 2013). Despite this limitation, we used this data given its harmonized data coverage for our study area.

Second, we measured total carbon footprint as the amount of CO_2_ released into the atmosphere due to human activities, which is estimated based on total population and their household expenditures (Moran et al., 2018). Data for per capita GDP and total carbon footprint are in the gridded format, and we summarized these indicators for each city by the area-mean (for per capita GDP) and sum (for total carbon footprint).

Third, we used SEI, which is the sum of standardized values of the proportion of households with access to water and a sewage system in the dwelling, the proportion of households that are not overcrowded, and the proportion of the population aged 25 years or above that completed primary education. Therefore, higher SEI values indicate more desirable housing, infrastructure and education conditions (Bilal et al., 2021).

Lastly, we introduced remotely sensed NTL, which is another widely used proxy of economic activities (Bennett & Smith, 2017). We measured area-mean of annual average NTL intensity for each city using the gridded VIIRS Stray Light Corrected Nighttime Day/Night Band Composites Version 1 dataset between 2014 and 2022. We expected NTL to be correlated with LST_night_ given the collection time of the dataset being close to midnight (1:30).

Both per capita GDP and SEI were available by administrative boundaries, whereas total carbon footprint and NTL were measured by the main urban cluster (Table S1). In our sample, the four economic indicators were weakly correlated with each other, with correlations between 0.162 and 0.514 (Spearman’s rank correlation), suggesting that they described different aspects of economic development (Fig. S1).

**Urban green space as mediators.** We introduced measures of urban green space to address the second research question on mediating effect. We used Normalized Difference Vegetation Index (NDVI), commonly referred as greenness in the literature, as a proxy for the quantity and vigor of urban green space. We obtained NDVI from the gridded MODIS daily satellite products at 250 m resolution (MOD13Q1.006). For each city-year observation, we calculated its greenness as the area-median of pixel-wise (i.e. per grid cell) annual maximum NDVI. We used pixel-wise annual maximum NDVI to capture the largest extent of urban green space, regardless of seasonality or different vegetation types. Similar to temperature, the end product here was city-specific time series of greenness, and we calculated the baseline and trend (Sen’s slope) of these time series as specific measures for green space. Baseline greenness measures the quantity and growth conditions of green space as of 2001, the starting year of the analysis, and greenness trend (i.e., greening) measures the changes in green space quantity and growth conditions between 2001 and 2022.

**Fig. 2:**
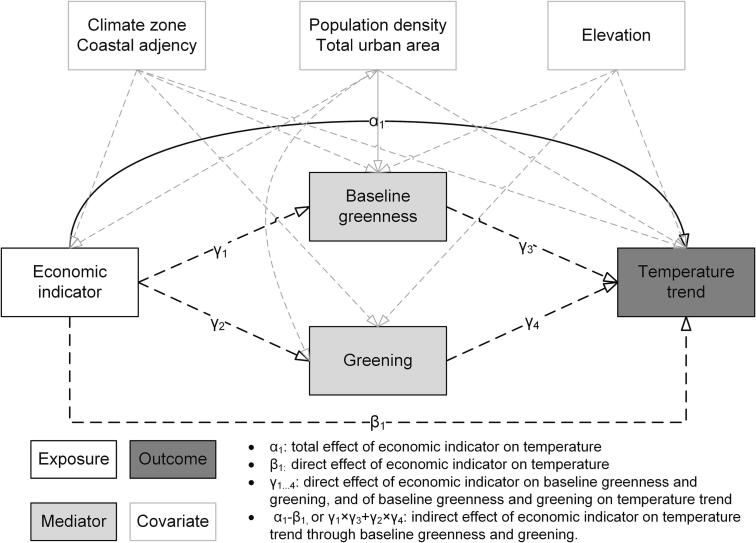
Directed acyclic graph (DAG) or path diagram illustrating the relationships between economic indicators (exposure), temperature trends (outcome), baseline greenness and greening (mediators), and covariates. Total effect measures the overall association between economic indicators and warming, including the direct effect and the indirect effect through green space. The direct effect is the association between economic indicators and warming, excluding the indirect effect through green space. The indirect effect represents the association between economic indicators and warming transmitted through green space.

**Covariates.** We included covariates for climate zones, elevation, coastal adjacency, population density, and total built up area, which may confound the relationship between temperature trend, economic indicators, baseline greenness, and greening. We selected these covariates using a directed acyclic graph (DAG, Fig. 2), which is a common approach for variable selection.

Climate zones influence local temperature and conditions of green space, and at the same time they may influence economic conditions, therefore qualifying as a confounder. For similar reasons, we controlled for average city elevation, adjacency to coast, population density, and total urban area. We obtained climate zones of the cities from Köppen-Geiger classification (Peel et al., 2007). Köppen-Geiger classification data is in gridded format, and we assigned each city to the climate zone that occupied most of the city’s area. Together, our study area contained tropical, temperate, arid, and polar climates. We calculated average elevation per city using the 30 m resolution Shuttle Radar Topography Mission (SRTM) dataset (Farr et al., 2007). Moreover, we derived total urban area by counting all the urbanized grid cells from a revised version of Global Urban Footprint (GUF) dataset (Esch et al., 2018). The revision, conducted by the SALURBAL project, assigned open and green space enclosed by GUF urban footprint as “urban” to create more continuous urban areas. By normalizing total population by the total urban area calculated earlier, the SAULRBAL project estimated city-level population density (Quistberg et al., 2018). Lastly, coastal adjacency was determined as whether a city had its boundary within 1 km from the coastline outlined by the Global Self-consistent, Hierarchical, High-resolution Geography Database (NOAA National Centers for Environmental Information, 2017, Wessel and Smith, 1996).

The data sources and their timeframes and spatial units of measurement for the outcome, exposures, mediators, and covariates are in Table S1. Prior to regression modeling, we examined descriptively the Spearman’s rank correlations among the temperature measures, economic indicators, greenness, and greening.

### 2.4 Path analysis with structural equation modeling

**Model setup**. We built a series of structural equation models (SEMs) and used path analysis to disentangle the relationships between temperature trends, economic indicators, baseline greenness, and greening. To avoid potential mediation and multicollinearity among the economic indicators, we investigated each indicator separately by including one indicator at a time in the model. These models were cross-sectional due to the constraints in obtaining longitudinal economic data. The basic structure of these SEMs is illustrated in the DAG (Fig. 2), where we hypothesized that some of the effect of economic indicators on temperature trends occurred through mediators including baseline greenness and greening. These models controlled for the covariates in section 2.3 and country-fixed effects to account for the differences between countries. In addition, we estimated country-cluster-robust standard errors to accounts for the autocorrelation among cities within the same country. We used chi-square, root mean square error of approximation (RMSEA), standardized root mean squared residual (SRMR), and comparative fit index (CFI) to calculate model fit of SEMs (Hooper et al., 2007). We fitted the models using the R package lavaan (Rosseel, 2012).

We interpreted the results of path analysis using the total, direct, and indirect effect of economic indicators on temperature trends. Total effect ($\alpha_{1}$) measures the overall association between economic indicators and warming, including the direct effect and the indirect effect through green space. The direct effect ($\beta_{1}$) is the association between economic indicators and warming, excluding the indirect effect through green space. The model also estimated the associations (direct effect) of economic indicators with baseline greenness ($\gamma_{1}$) and greening ($\gamma_{2}$), and those of baseline greenness and greening with the temperature trends ($\gamma_{3}$ and $\gamma_{4}$). The indirect effect ($\alpha_{1}-\beta_{1}$, or $\gamma_{1}\times \gamma_{3}+\gamma_{2}\times \gamma_{4}$) represent the association between economic indicators and warming transmitted through green space (Fig. 2).

**Robustness checks**. To test if the associations identified with our full sample varied by types of cities, we performed stratified analyses by economic conditions (top versus bottom 50% for a given economic indicator) and climate zones (arid versus non-arid) of the cities. These additional analyses are supported by the evidence that more economically developed cities may experience a slower warming rate following an inverted U shape of Environmental Kuznets Curve (Grossman & Krueger, 1995), that economically developed cities are resourceful to implement urban greening programs (Zhang et al., 2022), and that green space is sparser and more unevenly distributed in arid cities (known as the “luxury effect”, see Leong et al., 2018).

## 3 Results

### 3.1 Sample characteristics

A substantial portion (92% by T_air_, 75% by LST_day_, and 85% by LST_night_) of the 359 cities in our analysis experienced warming between 2001 and 2022. The average rate of warming was 0.221 °C/decade (interquartile range, IQR: [0.121, 0.335]) by T_air_, 0.199 °C/decade (IQR: [0.005, 0.430]) by LST_day_, and 0.337 °C/decade (IQR: [0.120, 0.558]) by LST_night_ (Table S2). In addition, there were weak correlations between the three temperature trends, measured by Spearman’s rank correlation. The strongest correlation of 0.519 was between trends of T_air_ and LST_night_, followed by 0.097 between trends of LST_day_ and LST_night_, and −0.026 between trends of T_air_ and LST_day_. Furthermore, these temperature trends were weakly correlated with the four economic indicators, with correlation coefficients between −0.168 and 0.210 (Fig. S1).

Between 2001 and 2022, 88% of cities experienced a decrease in greenness over time (browning). Greening on average was −0.029/decade (IQR: [-0.043, −0.014], Table S2), and it had weak and inconsistent correlations with economic indicators (per capita GDP: r = 0.255; total carbon footprint, r = -0.012; SEI, r = 0.237; NTL, r = 0.023) (Fig. S1). In addition, baseline greenness (mean: 0.545, IQR: [0.514, 0.623]) showed negative correlations between −0.209 and −0.408 with the economic indicators. The temperature trends had weak to moderate, negative correlations with baseline greenness and greening (i.e., more greenness or greening, less warming), except for the positive correlation between LST_day_ trend and baseline greenness. The correlations between temperature trends and baseline greenness were between −0.321 and 0.138, whereas the correlations between temperature trends and greening were between −0.006 and −0.304 (Fig. S1).

### 3.2 Associations between economic conditions and warming

**Fig. 3:**
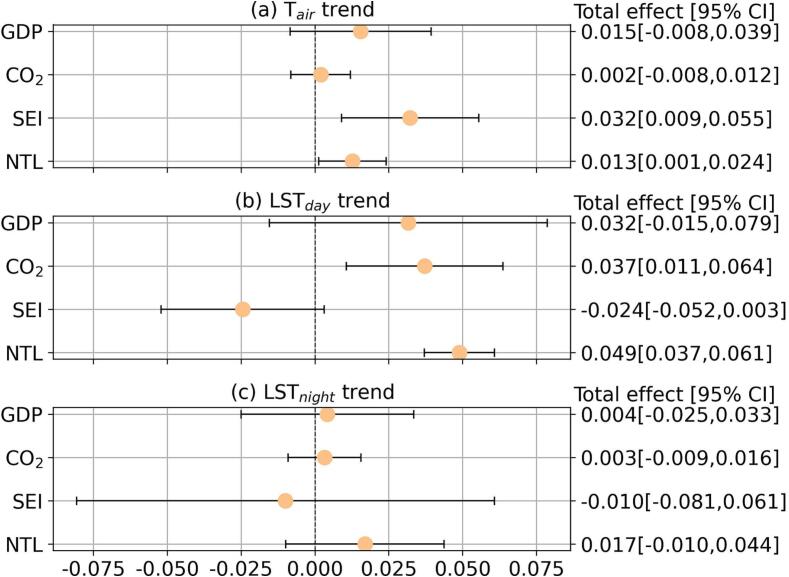
Total effects of economic conditions on temperature trends. Total effects (represented by the markers) are changes in temperature trend (°C/decade) for a one-standard-deviation increase in a corresponding economic indicator, when holding other covariates constant and factoring into the portion of changes transmitted through baseline greenness and greening. Models are adjusted for climate zone, coastal adjacency, land elevation, population density, total urban area, and country-fixed effects. 95% confidence intervals are marked by the error bars. Estimates for the total effects are also reported in Table S4. GDP: per capita GDP; CO_2_: total carbon footprint; SEI: Social Environment Index; NTL: nighttime light intensity.

**Fig. 4:**
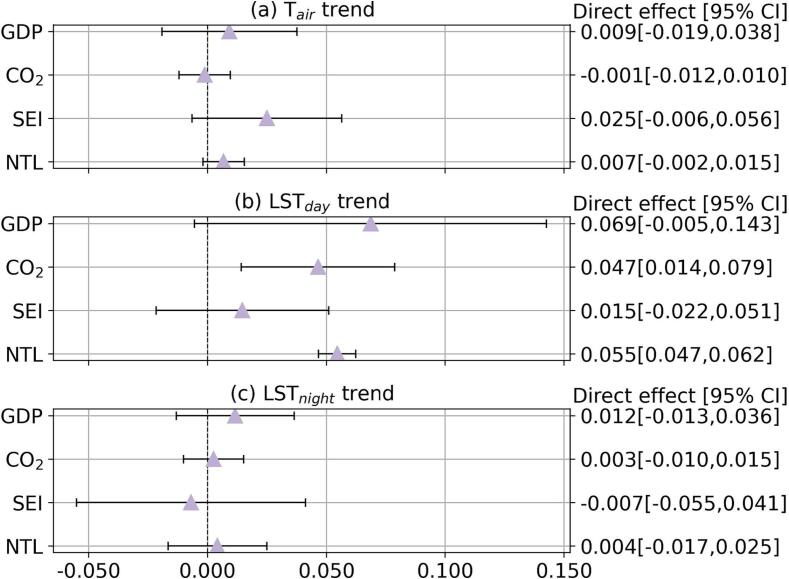
Direct effects of economic conditions on temperature trends. Direct effects (represented by the markers) are changes in temperature trend (°C/decade) for a one-standard-deviation increase in a corresponding economic indicator, when holding other covariates constant and excluding the proportion of changes transmitted through baseline greenness and greening. Models are adjusted for baseline greenness, greening, climate zone, coastal adjacency, land elevation, population density, total urban area, and country-fixed effects. 95% confidence intervals are marked by the error bars. Estimates for the direct effects are also reported in Table S4. GDP: per capita GDP; CO_2_: total carbon footprint; SEI: Social Environment Index; NTL: nighttime light intensity.

**Fig. 5:**
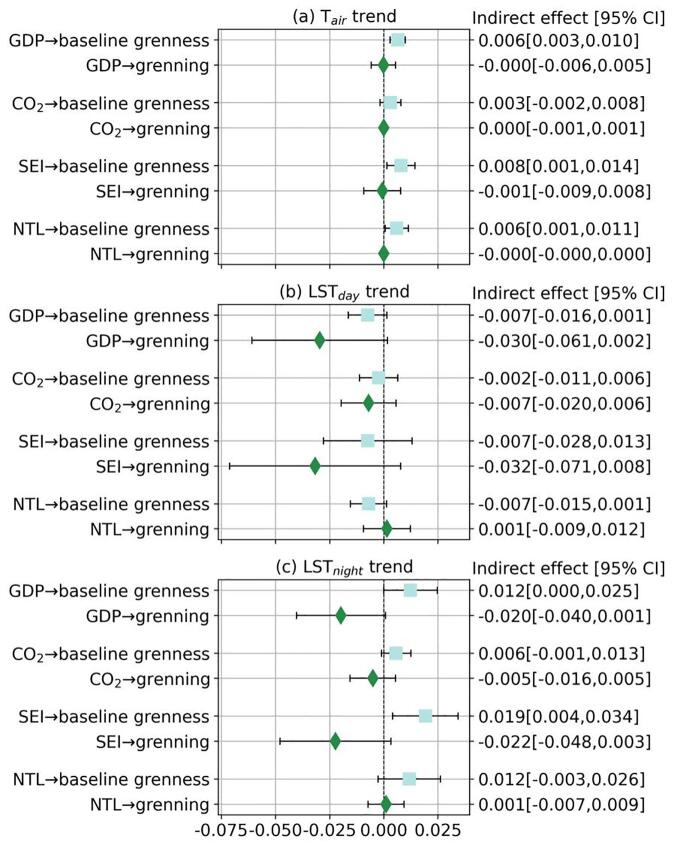
Indirect effects of economic conditions on temperature trends through baseline greenness and greening. Indirect effects (represented by the markers) are changes in temperature trend (°C/decade) for a one-standard-deviation increase in a corresponding economic indicator transmitted through baseline greenness and greening, when holding other covariates constant. Models are adjusted for economic conditions, climate zone, coastal adjacency, land elevation, population density, total urban area, and country-fixed effects. 95% confidence intervals are marked by the error bars. Estimates for the indirect effects are also reported in Table S7. GDP: per capita GDP; CO_2_: total carbon footprint; SEI: Social Environment Index; NTL: nighttime light intensity.

The structural equation models (SEMs) generally had satisfactory goodness-of-fit, with all SRMRs less than 0.08, and most CFIs greater than 0.96 (Table S3), consistent with the recommendations by Hu & Bentler (1999). We reported but did not solely rely on chi-square to determine model fit, as chi-square can be upwardly biased in models with many variables and small number of observations (n = 359) like this study (Shi et al., 2019). SEM estimates the total, direct, and indirect effects altogether, and as a robustness check for model fit we estimated these effects separately using ordinary least squares (OLS) models. The estimated effects from OLS models were similar to the ones from SEM. The stratified models for cities in arid climate have a poorer model fit, but we kept these models given their satisfactory SRMR values (Table S3) and to compare with other models. We represented the estimated model coefficients with forest plots for comparison purpose (Fig. 3, Fig. 4, Fig. 5), and we supplied path diagrams with the estimated model coefficients in the Supplementary Material (Fig. S2). Detailed model coefficient estimates can also be found in Tables S4-S7.

Our path analysis revealed some evidence that economic indicators were associated positively with warming based on the total effect (i.e., the association between an economic indicator and temperature trends including the proportion of association transmitted through baseline greenness and greening). There were statistically significant and positive total effects of SEI and NTL on T_air_ trend, with a one-standard-deviation (1-SD) increase in city-level SEI and NTL associated with 0.032 °C/decade (95% confidence interval, CI: [0.009,0.055]) and 0.013 °C/decade (95% CI: [0.001,0.024]) higher T_air_ trend, respectively (Fig. 3(a)). In addition, a 1-SD increase in total carbon footprint and NTL was associated with 0.037 °C/decade (95% CI: [0.011,0.064]) and 0.049 °C/decade (95% CI: [0.037,0.061]) higher LST_day_ trend, respectively (Fig. 3(b)). The total effects of economic indicators on LST_night_ trends were statistically non-significant (Fig. 3(c)).

Focusing on the direct effect of economic indicators on warming (i.e., the association between an economic indicator and temperature trends excluding the portion of association transmitted through baseline greenness and greening), we found a few statistically significant associations between economic indicators and LST_day_ trend (Fig. 4 (b)). The direct effects of economic indicators on other temperature trends were statistically non-significant (Fig. 4 (a, c)). Total carbon footprint and NTL showed statistically significant and positive direct effects on LST_day_ trend, with a 1-SD increase in total carbon footprint and NTL associated with 0.047 °C/decade (95% CI: [0.014,0.079]) and 0.055 °C/decade (95% CI: [0.047,0.062]) higher LST_day_ trend, respectively (Fig. 4 (b)).

### 3.3 Indirect effects through urban green space

City-wide economic conditions correlated negatively with baseline greenness but positively with greening to a lesser extent (Table S5). Furthermore, baseline greenness and greening correlated negatively with different temperature trends (Table S6). We found negative direct effects of per capita GDP, SEI, and NTL on baseline greenness, while the direct effect of total carbon footprint on baseline greenness were statistically non-significant. The direct effects of per capita GDP on greening were positive, whereas the direct effects of other economic indicators were statistically non-significant (Table S5). There were statistically significant, negative associations between baseline greenness and trends of T_air_ and LST_night_, but no statistically significant associations between baseline greenness and LST_day_ trend (Table S6). Greening was negatively and significantly associated with trends of LST_day_ and LST_night_, but its associations with T_air_ trend was statistically non-significant (Table S6).

The individual direct effects between economic conditions, green space, and temperature trends together lead to some statistical evidence that better economic conditions contributed to faster warming by lowering baseline greenness. Indirect effects of economic conditions on temperature trends through baseline greenness were statistically significant and positive in several paths, including the paths of “per capita GDP → T_air_ trend”, “SEI → T_air_ trend” and “NTL → T_air_ trend” (Fig. 5(a)), and the paths of “per capita GDP → LST_night_ trend” and “SEI → LST_night_ trend” (Fig. 5(c)). However, the indirect effects through greening were statistically non-significant in all the paths examined (Fig. 5), likely due to the statistically uncertain associations between economic conditions and greening (Table S5). Detailed estimates of these indirect effects can also be found in Table S7.

### 3.4 Stratified analysis by development levels and climate zones

The stratified analysis by economic development levels found that the total and direct effects of economic conditions on temperature trends estimated separately with more and less developed cities (top versus bottom 50% by a given economic indicator) were generally consistent with findings from the main analysis, namely, better economic conditions were associated with faster warming (Fig. S3). These findings were also supported by the stratified analysis by arid and non-arid cities (Fig. S4). The only exceptions were the negative total and direct effects of SEI on LST_day_ trend in arid cities (Fig. S4(c)).

The positive indirect effect of economic conditions on temperature trends through baseline greenness identified in the main analysis was supported in several cases by the stratified analysis (Fig. S3(a, c, g, h), Fig. S4(a, c, d, g)). A few exceptions were also observed. First, in less developed cities, the indirect effect through baseline greenness in the paths of “SEI → LST_day_ trend” and “NTL → LST_day_ trend” was negative (Fig. S3(g, h)), which was due to the positive association between baseline greenness and LST_day_ trend. Positive association between baseline greenness and LST_day_ trend was also found in non-arid cities, which similarly caused negative indirect effects of economic conditions on LST_day_ trend through baseline greenness (Fig. S4(e, g, h)). Second, in arid cities, we found positive associations between total carbon footprint and baseline greenness, which in turn lead to negative indirect effects through baseline greenness in the paths of “total carbon footprint → T_air_ trend” and “total carbon footprint → LST_night_ trend” (Fig. S4(b)).

Furthermore, the stratified analysis provided statistical support for a negative indirect effect through greening. In more developed cities, we found negative indirect effects through greening in paths involving per capita GDP (Fig. S3(a)). Negative indirect effect through greening was also identified for the less developed cities in the “per capita GDP → LST_day_ trend” path (Fig. S3(e)). Surprisingly, with less developed cities, the indirect effect through greening in the “NTL → LST_day_ trend” path was positive (Fig. S3(h)), resulting from negative association between NTL and greening. The negative indirect effect through greening was also evident in arid cities, with statistically significant ones in the paths of “per capita GDP → LST_day_ trend”, “per capita GDP → LST_night_ trend” (Fig. S4(a)), “SEI → LST_night_ trend” (Fig. S4(c)), and “NTL → LST_night_ trend” (Fig. S4(d)). However, none of this indirect effect through greening were statistically significant in non-arid cities.

## 4 Discussion

**Fig. 6:**
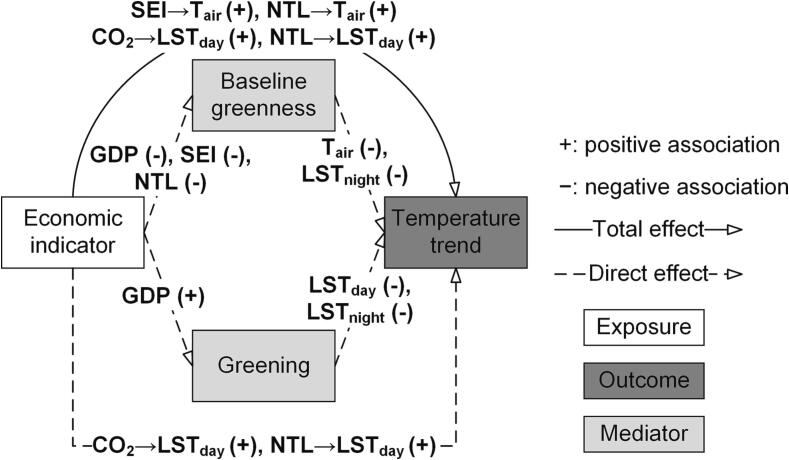
Summary of the relationships between economic conditions and urban warming, and the mediating role of baseline greenness and greening. Only statistically significant relationships are highlighted. Refer to Fig. 3, Fig. 4, Fig. 5 and Tables S4–S7 for detailed model estimates. GDP: per capita GDP; CO_2_: total carbon footprint; SEI: Social Environment Index; NTL: nighttime light intensity.

Regulating urban warming to improve human well-being urgently requires insights into how the well-studied physical drivers (e.g. changes in green space) are governed by less studied socioeconomic factors (Drescher, 2019, Jenerette et al., 2006). Our analysis based on 359 major Latin American cities in 10 countries quantifies the relationship between urban warming (i.e., increases in temperature over time) and multiple economic indicators, and evaluates the mediating role of urban green space in this relationship (summarized in Fig. 6). More specifically, we find that the nature and strength of associations between urban warming and economic conditions and mediation through urban green space vary by the economic indicator and temperature measure, affecting their interpretation (Fig. 3, Fig. 4, Fig. 5, also Fig. S3-S4 and Table S4-S7).

### 4.1 Contributions of economic conditions to urban warming

The positive total and direct effects of economic conditions on temperature trends suggest that better economic conditions may contribute to faster warming. SEI and NTL had positive total effects on T_air_ trend, and total carbon footprint and NTL showed positive total and direct effects on LST_day_ trend (Fig. 3, Fig. 4, Fig. 6 and Table S4). Total carbon footprint reflects the scale of human and economic activities, and it is expected to contribute positively to urban warming, similar to earlier findings on the positive association between city size, city-wide GDP and urban heat island effect (Y. Li et al., 2020). By construct, cities with higher SEI tend to have better housing and infrastructure conditions (Bilal et al., 2021), which may at the same time have a higher prevalence of heat-absorbing construction materials (e.g. pavement) that exacerbate warming (Myint et al., 2013). While improved housing conditions reflected by higher SEI may at the same time improve building energy efficiency, we only observed negative associations between SEI and LST_day_ trend in arid cities. NTL reflects human and economic activities particularly during the nighttime (Bennett & Smith, 2017), which was expected to contribute to greater nighttime warming. Interestingly, we did not find statistically significant total or direct effects of NTL on LST_night_ trend, while these effects on T_air_ and LST_day_ trends were statistically significant.

Positive associations between economic conditions and temperature trends observed in this study differ from some prior research findings. For example, one study based on Chinese cities found that per capita GDP was negatively associated with urban heat island effect, and the authors speculated that lower energy use per unit of GDP might alleviate urban warming (Y. Li et al., 2020). It is also plausible that more developed cities may adopt energy efficiency programs in architecture and transportation to curb urban warming (Núñez Collado and Wang, 2020, Rehermann and Pablo-Romero, 2018). However, these strategies may not reverse the growing trend of energy use and heat releases due to growing demand (Rehermann & Pablo-Romero, 2018).

### 4.2 The mediation of green space on warming associated with economic conditions

The legacy loss of green space, reflected by lower baseline greenness as of year 2001 in cities with better economic conditions, exacerbates warming in T_air_ and LST_night_ attributable to economic development. Baseline greenness was negatively associated with economic indicators except total carbon footprint (Fig. 6 and Table S5), which, coupled with its salient cooling potential for T_air_ and LST_night_ trends (Fig. 6 and Table S6), suggests that baseline greenness mediates the association between economic conditions and warming (Fig. 5 and Table S7). The lack of green space in cities with better economic conditions has been reported in a handful of studies focusing on Latin America (Ju et al., 2021), China (G. Li et al., 2015, Sun et al., 2011), and the US (Browning & Rigolon, 2018), while the cooling effect of green space has been extensively studied (e.g. Ziter et al., 2019). Low baseline greenness here likely reflects legacy land cover changes in the course of economic development and urbanization prior to our study period (2001–2022), given that we excluded the differences in background environment by conditioning on several biophysical covariates. Therefore, our finding provides statistical evidence that improved economic conditions exacerbates urban warming measured by T_air_ and LST_night_ through a legacy of converting green space to other land covers. However, the negative indirect effect of baseline greenness on the associations between total carbon footprint and trends of T_air_ and LST_night_ in arid cities (Fig. S4(b)) were contrary to what was reported for the rest of the cities. This was due to the positive association between total carbon footprint and baseline greenness, a pattern suggesting that larger and more developed cities in arid climates might be more resourceful in maintaining green space, which has been reported in a few other studies (Schell et al., 2020, Zhang et al., 2022).

In contrast to earlier findings suggesting mediation of baseline greenness on warming of T_air_ and LST_night_ associated with economic conditions, such mediation was statistically non-significant for LST_day_ trend (Fig. 5 and Table S7). This was attributable to the non-significant associations between baseline greenness and LST_day_ trend, contrasting with the significant associations observed for T_air_ and LST_night_ trends (Fig. 6 and Table S6). A potential explanation could lie in the different physical nature of these temperatures: compared with T_air_ and LST_night_, LST_day_ trend is more sensitive to temporal changes in microclimate conditions, particularly those changes in photosynthetically active vegetation and non-vegetated surfaces over time (Nichol, 2005, Oke, 1976). Since baseline greenness does not directly reflect such temporal changes, its uncertain association with LST_day_ trend may be expected. Conversely, the association is statistically significant between LST_day_ trend and greening that reflects temporal changes in green space (Fig. 6 and Table S6). However, we identified statistically significant and positive association between baseline greenness and LST_day_ trend in less developed and non-arid cities, contrary to the expected cooling effect. The omitted factors driving this unexpected association require further investigation.

We found a statistically non-significant mediating effect of greening on the association between economic conditions and warming using the entire sample of 359 cities. Despite the significant cooling effect of greening on both LST trends (Fig. 6 and Table S6), we found limited statistically evidence that greening was associated with economic conditions (Fig. 6 and Table S5), causing the mostly non-significant mediation. The mostly non-significant association between economic conditions and greening may also reflect our focus on the main urban cluster where land cover changes including greening is likely limited (Fig. 1(b)). However, the association between economic conditions and greening may become more evident when using alternative definitions of a “city”. For instance, we previously found that more developed Latin American cities defined by administrative boundaries experienced greater greening between 2000 and 2015 (Ju et al., 2021). In addition, we only found statistically significant cooling effect of greening on LST trends but not on T_air_ trend (Fig. 6 and Table S6), consistent with that LSTs were generally more sensitive to land cover changes than T_air_.

Despite the overall pattern being statistically non-significant, there was some statistical support for the negative mediating effect of greening on the relationship between economic conditions and warming among arid cities (Fig. S4(a, c, d), and less so for more economically developed cities (Fig. S3(a)). The finding suggested that cities of better economic conditions, particularly those in arid climate, are more likely to enjoy cooling from recent greening when compared with other cities. This adds evidence to the hypothesis of “luxury effect” and “deprivation amplification”, suggesting that cities of higher socioeconomic status tend to enjoy greater environmental resources and their ecosystem services (Macintyre, 2007, Schell et al., 2020).

### 4.3 Implications for urban planning and green space management

Together, our findings call for more equitable urban greening efforts in Latin American cities. Despite the well-documented cooling effect of green space and its promise as a heat-mitigating urban planning strategy, we confirmed that green space and its cooling potential were not evenly distributed between the cities of different economic conditions in Latin America. The negative association between economic conditions and baseline greenness can be explained by green space losses during early stages of urbanization and economic development. This loss of green space further exacerbates urban warming. While cities with better economic conditions may have the resources to maintain and restore their green space (Zhang et al., 2022), less developed cities may continue to convert green space to built-up areas to boost economic development and urbanization. The latter trend means that less developed cities will have a higher chance to lose potential cooling from green space, which in turn exacerbates their warming and lead to other consequences such as elevated heat-mortality risks (Kephart et al., 2022).

Urban greening programs should prioritize less developed cities for a few reasons. First, these cities likely face greater pressure to preserve green space from ongoing urbanization and often lack the resources for urban greening and other climate adaptation programs. Second, less developed cities may continue to experience urban warming from other heat sources, such as increased energy use and traffic volume in the course of economic development and urbanization. However, socioeconomic equity in urban greening programs is likely an overlooked agenda in Latin America, given the lack of planning, the complexity of its governance, and the entrenched inequalities in this region (Arantes et al., 2021, Fernandez et al., 2022).

### 4.4 Limitations and future research directions

Several limitations and future research directions should be addressed. First, due to limited temporal availability of LST data, we only included a 22-year window (2001–2022) in this study. Therefore, future studies should focus on air temperature that has longer historical records. Second, baseline greenness and greening based on remotely sensed vegetation indices did not explicitly convey type, biomass, structure, and landscape pattern of green space (Ju et al., 2021). This makes it hard to pinpoint more specific pathways connecting urban warming with economic conditions through green space. For example, if more economically developed cities have more fragmented green space, and to what degree this fragmentation contributes to warming. Future studies can address this shortcoming by employing more semantically explicit maps of urban green space, which are increasingly available (Huang et al., 2021). Another specific pathway worthy of investigation is the disparity in cooling efficiency of green space, which starts to be addressed by few studies focusing on the contrast between advantaged versus deprived neighborhoods in US cities (Lin et al., 2023) and between Global North and South cities (Y. Li et al., 2023). Third, economic data for the city samples were not available over an extended timeframe, limiting our ability to perform longitudinal analysis. A longitudinal analysis with time varying economic indicators is critical for identifying the causal effect of economic development on urban warming, which we investigated here through regression adjustment and path analysis but with cross-sectional data.

Furthermore, information on additional urban warming contributors (e.g., building energy use, transportation emissions, and urban design) may still be sparse. This limits further analyses on additional paths between economic conditions and warming, as well as fine-grained studies at neighborhood scale that are more relevant to decision-makers and stakeholders. Future studies could also adopt comprehensive indices describing the sustainability status of the city to examine whether current sustainability efforts curb urban warming and improve green space provisioning. Lastly, it is possible that some unobserved variables, such as urban design and planning, caused omitted variable bias in the associations identified in our analysis. This may explain some unexpected findings in this study, for example, the results showing positive associations between baseline greenness and LST_day_ trend in less developed and non-arid cities. These limitations also suggest that in addition to observational studies, research on this topic should consider process- and agent-based models on how different urban sectors (e.g. building energy use and transportation) and specific design/planning decisions (e.g. those on the provisioning and spatial configuration of green space) contribute to urban warming (Kong et al., 2016, McRae et al., 2020).

## 5 Conclusions

In this study, we investigated the relationship between urban warming (increases in temperature over time), economic conditions, and the provisioning and changes in urban green space using a sample of 359 major Latin American cities from 10 counties between the years of 2001 and 2012. We found that cities with better economic conditions were associated with faster urban warming. In addition, green space mediated the association between city-wide economic conditions and urban warming through two opposite paths. In one end, better economic conditions were associated with a legacy loss of green space and lowered baseline greenness as of year 2001, leading to faster warming. At the same time, there was modest evidence that more desirable economic conditions were associated with greater greening between 2001 and 2012, which led to cooling and partially curbs raising temperatures, especially in arid and more economically developed cities.

Together, these findings shed light on how green space regulates urban warming under the influence of economic conditions. While the cooling effect of green space is well recognized, green space itself may not always be available due to historical losses from development, inequitable land use practices, and segregation. The increased greening in more economically developed cities, although marginal as observed in this study, shows the potential to curb urban warming if such greening trend continues. Further attention should be paid to studying urban greening given its promising role to provide climate regulation and other ecosystem services, while addressing its potential socioeconomic disparities.

## Declaration of Competing Interest

The authors declare that they have no known competing financial interests or personal relationships that could have appeared to influence the work reported in this paper.

## Data Availability

Data will be made available on request.
