# Supplementary material for: Recent greening may curb urban warming in Latin American cities of better economic conditions
Source: Landsc Urban Plan. 2023 Dec;240:None. doi: 10.1016/j.landurbplan.2023.104896 (PMC10570748; doi:10.1016/j.landurbplan.2023.104896)
Supplement: Supplementary data 1 [file mmc1.pdf]

Supplementary Material

Recent greening curbs urban warming in Latin American cities of better economic conditions

Table of contents

Table S1 Data sources and time frame of the variables and spatial unit for their measurements........2

Table S2 Summary statistics........4

Table S3 Model goodness of fit........5

Table S4 Total and direct effect of socioeconomic indicators on temperature trends...7

Table S5 Direct effect of socioeconomic indicators on baseline greenness and greening........8

Table S6 Direct effect of baseline greenness and greening on temperature trends 9

Table S7 Indirect effect of socioeconomic indicators on temperature trends, through baseline greenness and greening........10

Figure S1 Spearman's rank correlations between temperature trends, baseline greenness (year 2001), greening (years 2001-2022), and socioeconomic indicators.. 11

Figure S2 Path diagrams showing direct and total effects of socioeconomic indicators on temperature trends........12

Figure S3 Stratified analysis by (a-c) more and (d-f) less developed cities, showing total, direct, and indirect effects of per capita GDP, total carbon footprint, and social environment index (SEI) on temperature trends........13

Figure S4 Stratified analysis by (a-c) arid and (d-f) non-arid cities, showing total, direct, and indirect effects of per capita GDP, total carbon footprint, and social environment index (SEI) on temperature trends........14

Table S1 Data sources and time frame of the variables and spatial unit for their measurements

**Variable**	**Time frame**	**Measured by which spatial unit?**	**Source/References**
Air Temperature	2001-2022	Main urban cluster	ERA5-Land (Copernicus Climate Change Service (C3S), 2019)
Daytime and nighttime land surface temperature	2001-2022	Main urban cluster	MODIS terra land surface temperature 8-day at 1km resolution (MOD11A2.061)
Per capita GDP	Averaged between 2001 and 2015	Administrative boundary	(Kummu et al., 2018)
Total carbon footprint	2013	Main urban cluster	(Moran et al., 2018)
Social Environment Index	Argentina (2010), Brazil (2010), Chile (2002), Colombia (2005), Costa Rica (2011), Guatemala (2002), Mexico (2010), Panama (2010), Peru (2007), El Salvador (2007)	Administrative boundary	(Bilal et al., 2021)
Nighttime light intensity	Averaged between 2014 and 2022	Main urban cluster	VIIRS Stray Light Corrected Nighttime Day/Night Band Composites Version 1
Baseline greenness	2001	Main urban cluster	MODIS daily satellite products at 250m resolution (MOD13Q1.006)
Greening	2001-2022		
Climate zone	Version of 1986-2010	Main urban cluster	Köppen Climate classification (Kottek et al., 2006)
Elevation	2000	Main urban cluster	Shuttle Radar Topography Mission (SRTM) (Farr et al., 2007)
Coastal adjacency	2017	Main urban cluster	Based on coastal lines from Global Self-consistent, Hierarchical, High-resolution

			Geography Database (GSHHG)(NOAA National Centers for Environmental Information, 2017; Wessel & Smith, 1996)
Population density	2010	Main urban cluster	The Salud Urbana en América Latina (SALURBAL; Urban Health in Latin America) project (Quistberg et al., 2018)
Total urban area	2011-2012 (93%) with missing observations filled by data from 2013-2014 (7%)	Main urban cluster	Global urban footprint (Esch et al., 2018)

Table S2 Summary statistics

Variable	Mean	Std. dev.	Min.	25th pctl.	Mdn.	75th pctl.	Max.
T_{air} trend (°C/decade)	0.221	0.153	-0.123	0.121	0.220	0.335	0.673
LST_{day} trend (°C/decade)	0.199	0.352	-1.206	0.005	0.228	0.430	1.195
LST_{night} trend (°C/decade)	0.337	0.309	-0.577	0.120	0.314	0.558	1.045
Per capita GDP(10,000 2011 US dollars)	1.380	0.790	0.259	0.861	1.235	1.741	8.140
Total carbon footprint (10^8 ton)	0.021	0.056	0.000	0.004	0.008	0.018	0.648
SEI	0.003	0.642	-2.182	-0.320	0.122	0.493	1.081
NTL (nanoWatts/ cm^2/sr)	29.512	14.001	5.818	21.476	27.241	34.255	146.824
Baseline greenness	0.545	0.131	0.106	0.514	0.574	0.623	0.815
Greening (/decade)	-0.029	0.024	-0.114	-0.043	-0.027	-0.014	0.030

Note: n = 359 cities; Baseline greenness is the greenness as of year 2001, and greening is the changes in greenness per decade between 2001 and 2022; Pctl.: percentile; Mdn.: median; SEI: Social Environment Index; NTL: nighttime light intensity.

Table S3 Model goodness of fit

Variable	T_{air} trend				LST_{day} trend				LST_{night} trend			
	Chi-square	RMSEA	SRMR	CFI	Chi-square	RMSEA	SRMR	CFI	Chi-square	RMSEA	SRMR	CFI
**Model using all cities (n=359)**												
Per capita GDP	2.649	0.068 [0.044,0.092]	0.014	0.998	2.649	0.068 [0.044,0.092]	0.014	0.999	2.649	0.068 [0.044,0.092]	0.014	0.998
Total carbon footprint	3.925**	0.090 [0.066,0.116]	0.017	0.996	3.925**	0.090 [0.066,0.116]	0.017	0.997	3.925**	0.090 [0.066,0.116]	0.016	0.996
SEI	2.113	0.056 [0.032,0.079]	0.013	0.999	2.113	0.056 [0.032,0.079]	0.014	0.999	2.113	0.056 [0.032,0.079]	0.013	0.999
NTL	4.735**	0.102 [0.077,0.128]	0.017	0.996	4.735**	0.102 [0.077,0.128]	0.017	0.997	4.735**	0.102 [0.077,0.128]	0.017	0.995
**Model using more developed cities (n=183^{+}, $179^{\wedge}$, or $180^{\#}$)**												
Per capita GDP	1.699	0.065 [0.029,0.098]	0.021	0.999	1.699	0.065 [0.029,0.098]	0.020	0.999	1.699	0.065 [0.029,0.098]	0.021	0.999
Total carbon footprint	3.562*	0.120 [0.077,0.165]	0.020	0.993	3.562*	0.120 [0.077,0.165]	0.021	0.995	3.562*	0.120 [0.077,0.165]	0.020	0.993
SEI	3.166*	0.110 [0.076,0.146]	0.023	0.997	3.166*	0.110 [0.076,0.146]	0.021	0.998	3.166*	0.110 [0.076,0.146]	0.021	0.997
NTL	7.607***	0.192 [0.150,0.237]	0.031	0.979	7.607***	0.192 [0.150,0.237]	0.031	0.987	7.607***	0.192 [0.150,0.237]	0.030	0.983
**Model using less developed cities (n=176^{+}, $180^{\wedge}$, or $179^{\#}$)**												
Per capita GDP	6.837***	0.173 [0.098,0.259]	0.012	0.988	6.836***	0.173 [0.098,0.259]	0.012	0.991	6.836***	0.173 [0.098,0.259]	0.011	0.988
Total carbon footprint	3.682*	0.122 [0.075,0.173]	0.014	0.997	3.682*	0.122 [0.075,0.173]	0.013	0.997	3.682*	0.122 [0.075,0.173]	0.013	0.997
SEI	1.539	0.055	0.012	0.999	1.539	0.055	0.012	0.999	1.539	0.055	0.012	0.999

		[0.000,0.109]				[0.000,0.109]				[0.000,0.109]		
NTL	1.757	0.065 [0.000,0.122]	0.010	0.998	1.757	0.065 [0.000,0.122]	0.010	0.999	1.757	0.065 [0.000,0.122]	0.010	0.998
**Model using arid cities (n=78)**												
Per capita GDP	23.192***	0.533 [0.392,0.689]	0.044	0.938	23.192***	0.533 [0.392,0.689]	0.044	0.969	23.192***	0.533 [0.392,0.689]	0.043	0.942
Total carbon footprint	74.853***	0.973 [0.751,1.216]	0.055	0.782	74.853***	0.973 [0.751,1.216]	0.055	0.869	74.853***	0.973 [0.751,1.216]	0.055	0.783
SEI	20.695***	0.502 [0.382,0.634]	0.049	0.949	20.695***	0.502 [0.382,0.634]	0.046	0.963	20.695***	0.502 [0.382,0.634]	0.047	0.943
NTL	43.515***	0.738 [0.567,0.926]	0.056	0.849	43.515***	0.738 [0.567,0.926]	0.056	0.901	43.515***	0.738 [0.567,0.926]	0.055	0.846
**Model using non-arid cities (n=281)**												
Per capita GDP	3.535*	0.095 [0.013,0.190]	0.008	0.995	3.535*	0.095 [0.013,0.190]	0.008	0.996	3.535*	0.095 [0.013,0.190]	0.007	0.993
Total carbon footprint	5.082**	0.121 [0.038,0.226]	0.008	0.992	5.082**	0.121 [0.038,0.226]	0.008	0.995	5.082**	0.121 [0.038,0.226]	0.008	0.990
SEI	1.443	0.040 [0.000,0.126]	0.006	0.999	1.443	0.040 [0.000,0.126]	0.006	0.999	1.443	0.040 [0.000,0.126]	0.006	0.999
NTL	17.588***	0.243 [0.122,0.394]	0.010	0.968	17.588***	0.243 [0.122,0.394]	0.010	0.979	17.588***	0.243 [0.122,0.394]	0.009	0.959

Note: RMSEA: root mean square error of approximation; SRMR: standardized root mean squared residual; CFI: comparative fit index. SEI: Social Environment Index; NTL: nighttime light intensity. 90% confidence interval of RMSEA are reported in the square brackets. According to Hu & Bentler (1999), a properly fitted model has a CFI of 0.96 or higher and a SRMR of 0.09 or lower. We reported but did not solely rely on chi-square to determine model fit, as chi-square can be upwardly biased in models with many variables and small number of observations like the ones in this study (Shi et al., 2019).

[+]: number of cities stratified by per capita GDP, [^]: number of cities stratified by total carbon footprint and SEI, [#]: number of cities stratified by NTL. * indicate significant at p-value < 0.10, ** indicate significant at p-value < 0.05, and *** indicate significant at p-value < 0.01.

Table S4 Total and direct effect of socioeconomic indicators on temperature trends

Variable	T_{air} trend (°C/decade)		LST_{day} trend (°C/decade)		LST_{night} trend (°C/decade)	
	**total effect**	**direct effect**	**total effect**	**direct effect**	**total effect**	**direct effect**
Per capita GDP	0.015 [-0.008, 0.039]	0.009 [-0.019, 0.038]	0.032 [-0.015, 0.079]	0.069 [-0.005, 0.143]	0.004 [-0.025, 0.033]	0.012 [-0.013, 0.036]
Total carbon footprint	0.002 [-0.008, 0.012]	-0.001 [-0.012, 0.010]	0.037*** [0.011, 0.064]	0.047*** [0.014, 0.079]	0.003 [-0.009, 0.016]	0.003 [-0.010, 0.015]
SEI	0.032*** [0.009, 0.055]	0.025 [-0.006, 0.056]	-0.024 [-0.052, 0.003]	0.015 [-0.022, 0.051]	-0.010 [-0.081, 0.061]	-0.007 [-0.055, 0.041]
NTL	0.013** [0.001, 0.024]	0.007 [-0.002, 0.015]	0.049*** [0.037, 0.061]	0.055*** [0.047, 0.062]	0.017 [-0.010, 0.044]	0.004 [-0.017, 0.025]

Note: A coefficient represents the changes in temperature trend (+/- °C/decade) for a one-standard-deviation increase in a socioeconomic indicator, measured by total effect and direct effect while holding other covariates constant. A path diagram of the models is in Figure 2. Models estimating total effect are adjusted for covariates including climate zone, coastal adjacency, land elevation, population density, total urban area, and country-fixed effects. Models estimating direct effect additionally control for baseline greenness (year 2001), and greening (years 2001-2022). The coefficients for the covariates adjusted are not shown for display purpose. ** and *** indicate significant at p-value < 0.05 and p-value <0.01. 95% confidence intervals are in square brackets. SEI: Social Environment Index; NTL: nighttime light intensity.

Table S5 Direct effect of socioeconomic indicators on baseline greenness and greening

Variable	Baseline greenness	Greening
Per capita GDP	-0.188***<br>[-0.274, -0.101]	0.264**<br>[0.050, 0.477]
Total carbon footprint	-0.084<br>[-0.202, 0.034]	0.069<br>[-0.052, 0.190]
SEI	-0.272***<br>[-0.387, -0.158]	0.308<br>[-0.047, 0.664]
NTL	-0.174**<br>[-0.305, -0.042]	-0.015<br>[-0.128, 0.099]

Note: A coefficient represents the number of standard deviations that baseline greenness (year 2001) and greening (years 2001-2022) changes for a one-standard-deviation increase a socioeconomic indicator, measured by direct effect and holding other covariates constant. A path diagram of the models is in Figure 2. Models are adjusted for climate zone, coastal adjacency, land elevation, population density, total urban area, and country-fixed effects. The coefficients for the covariates adjusted are not shown for display purpose. ** and *** indicate significant at p-value < 0.05 and p-value <0.01. 95% confidence intervals are in square brackets. SEI: Social Environment Index; NTL: nighttime light intensity.

Table S6 Direct effect of baseline greenness and greening on temperature trends

**Path**	**T_{air} trend (°C/decade)**	**LST_{day} trend (°C/decade)**	**LST_{night} trend (°C/decade)**
Per capita GDP→ baseline greenness→ T trend	-0.034*** [-0.052, -0.016]	0.040 [-0.015, 0.094]	-0.066*** [-0.111, -0.021]
Per capita GDP → greening→ T trend	-0.001 [-0.022, 0.020]	-0.112*** [-0.145, -0.079]	-0.075*** [-0.112, -0.038]
Total carbon footprint → baseline greenness → T trend	-0.037*** [-0.055, -0.019]	0.028 [-0.046, 0.102]	-0.068*** [-0.112, -0.024]
Total carbon footprint → greening→ T trend	0.001 [-0.015, 0.016]	-0.102*** [-0.119, -0.085]	-0.073*** [-0.109, -0.037]
SEI→ baseline greenness→ T trend	-0.029*** [-0.043, -0.015]	0.027 [-0.045, 0.099]	-0.071*** [-0.121, -0.020]
SEI→ greening → T trend	-0.002 [-0.028, 0.024]	-0.103*** [-0.124, -0.082]	-0.072*** [-0.107, -0.038]
NTL→ baseline greenness→ T trend	-0.034*** [-0.051, -0.018]	0.041 [-0.027, 0.108]	-0.067*** [-0.115, -0.019]
NTL→ greening → T trend	0.001 [-0.015, 0.017]	-0.096*** [-0.111, -0.080]	-0.073*** [-0.109, -0.037]

Note: A coefficient represents the changes in temperature trend (+/- °C/decade) for a one-standard-deviation increase in baseline greenness (year 2001) and greening (years 2001-2022), measured by direct effect and holding other covariates constant. A path diagram of the models is in Figure 2. Models are adjusted for socioeconomic indicators, climate zone, coastal adjacency, land elevation, population density, total urban area, and country-fixed effects. The coefficients for the covariates adjusted are not shown for display purpose. ** and *** indicate significant at p-value < 0.05 and p-value <0.01. 95% confidence intervals are in square brackets. SEI: Social Environment Index; NTL: nighttime light intensity; T: temperature.

Table S7 Indirect effect of socioeconomic indicators on temperature trends, through baseline greenness and greening

Path	T_{air} trend (°C/decade)	LST_{day} trend (°C/decade)	LST_{night} trend (°C/decade)
Per capita GDP→ baseline greenness→ T trend	0.006*** [0.003, 0.010]	-0.007 [-0.016, 0.001]	0.012** [0.000, 0.025]
Per capita GDP→ greening→ T trend	-0.000 [-0.006, 0.005]	-0.030 [-0.061, 0.002]	-0.020 [-0.040, 0.001]
Total carbon footprint → baseline greenness → T trend	0.003 [-0.002, 0.008]	-0.002 [-0.011, 0.006]	0.006 [-0.001, 0.013]
Total carbon footprint → greening→ T trend	0.000 [-0.001, 0.001]	-0.007 [-0.020, 0.006]	-0.005 [-0.016, 0.005]
SEI→ baseline greenness→ T trend	0.008** [0.001, 0.014]	-0.007 [-0.028, 0.013]	0.019** [0.004, 0.034]
SEI→ greening→ T trend	-0.001 [-0.009, 0.008]	-0.032 [-0.071, 0.008]	-0.022 [-0.048, 0.003]
NTL→ baseline greenness→ T trend	0.006** [0.001, 0.011]	-0.007 [-0.015, 0.001]	0.012 [-0.003, 0.026]
NTL→ greening→ T trend	-0.000 [-0.000, 0.000]	0.001 [-0.009, 0.012]	0.001 [-0.007, 0.009]

Note: A coefficient represents the changes in temperature trend (+/- °C/decade) for a one-standard-deviation increase in a socioeconomic indicator, measured by indirect effect through baseline greenness (year 2001) and greening (years 2001-2022), while holding other covariates constant. A path diagram of the models is in Figure 2. Models are adjusted for socioeconomic indicators, climate zone, coastal adjacency, land elevation, population density, total urban area, and country-fixed effects. The coefficients for the covariates adjusted for are not shown for display purpose. ** and *** indicate significant at p-value < 0.05 and p-value <0.01. 95% confidence intervals are in square brackets. SEI: Social Environment Index; NTL: nighttime light intensity; T: temperature.

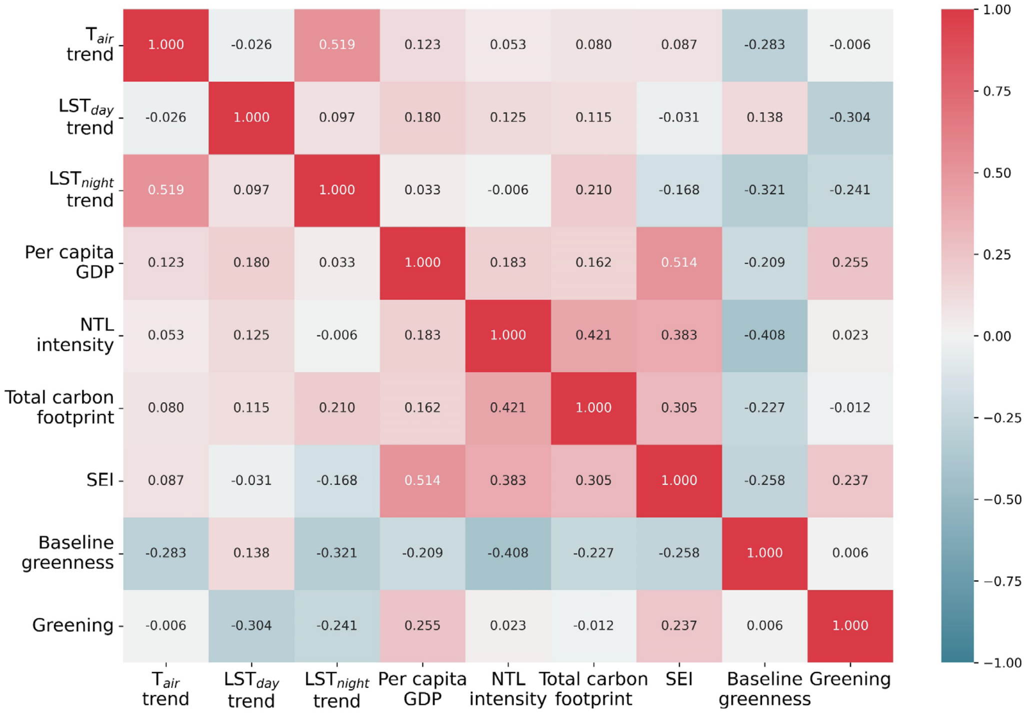

Figure S1 Spearman's rank correlations between temperature trends, baseline greenness (year 2001), greening (years 2001-2022), and socioeconomic indicators. SEI: Social Environment Index; NTL: nighttime light intensity.

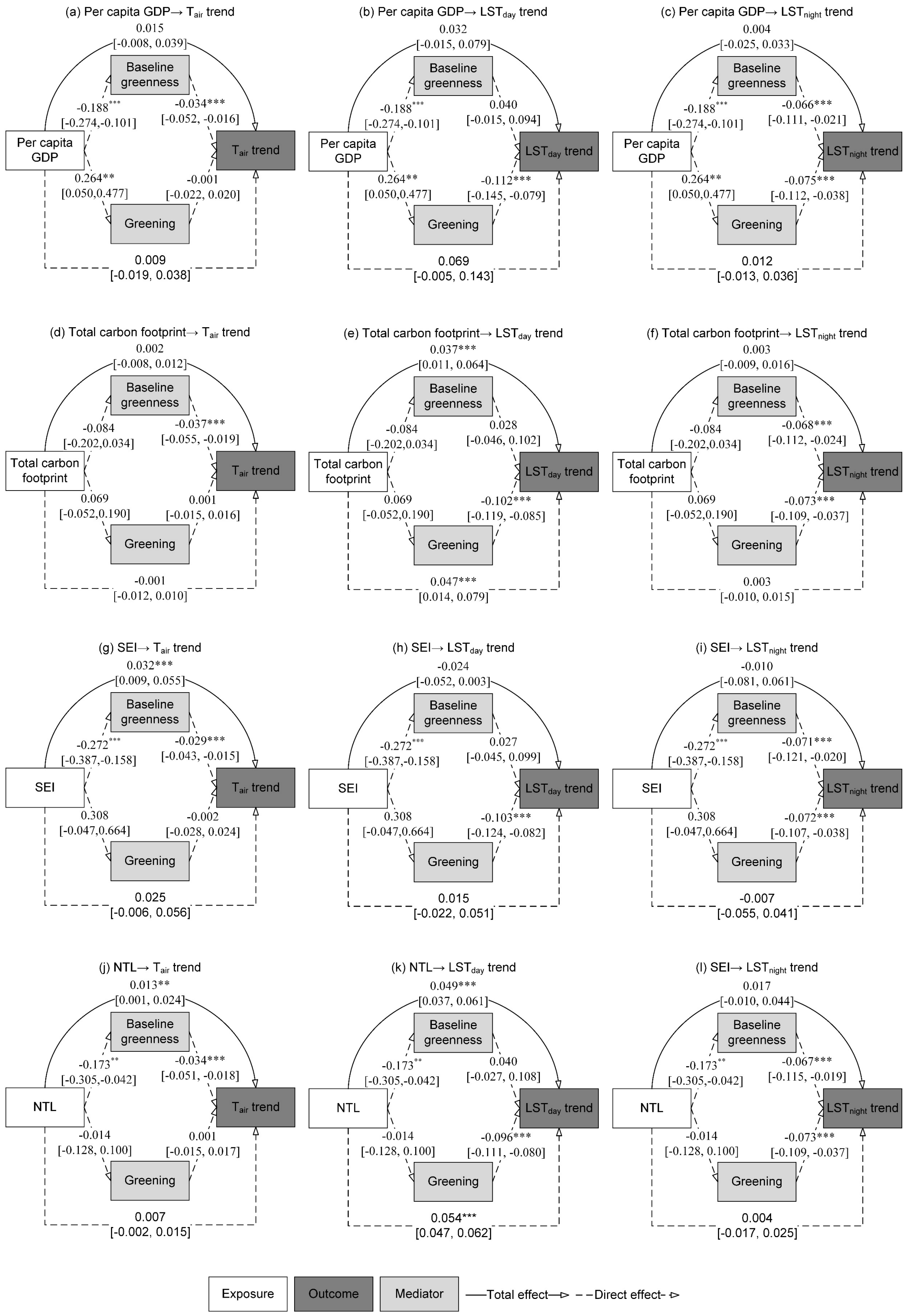

Figure S2 Path diagrams showing direct and total effects of socioeconomic indicators on temperature trends. ** and *** indicate significant at p-value < 0.05 and p-value <0.01. 95% confidence intervals are in square brackets. SEI: Social Environment Index; NTL: nighttime light intensity.

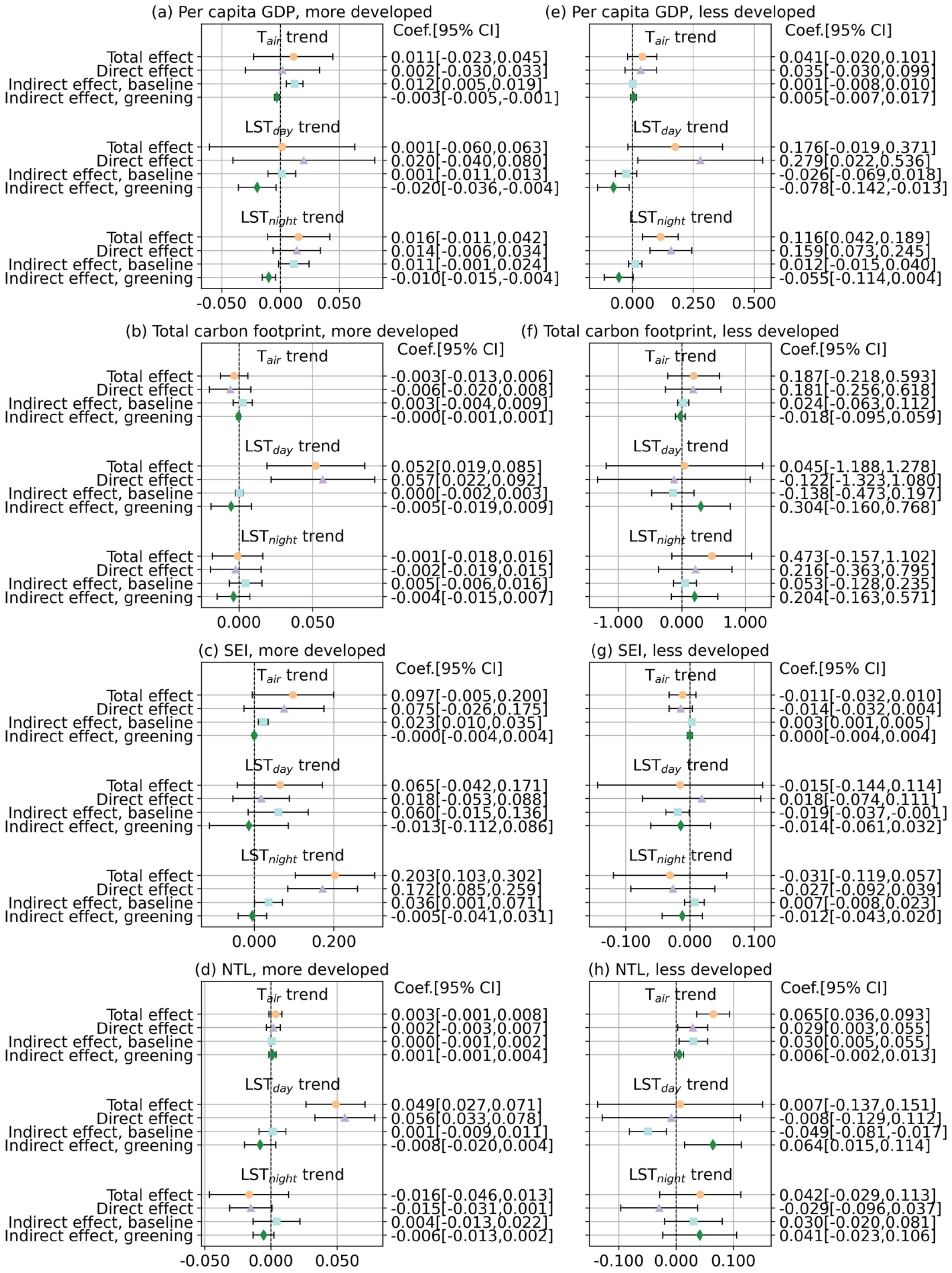

Figure S3 Stratified analysis by (a-d) more and (e-h) less developed cities, showing total, direct, and indirect effects of per capita GDP, total carbon footprint, social environment index (SEI), and nighttime light intensity (NTL) on temperature trends. More developed cities are those within the top 50% of a socioeconomic variable. The estimates represent changes in temperature trend (°C/decade) for a one-standard-deviation increase in the corresponding covariate, when holding other covariates constant. Models estimating total effect adjust for climate zone, coastal adjacency, land elevation, population density, total urban area, and country-fixed effects. Models estimating direct and indirect effects additionally control for temperature, greenness, and greening. 95% confidence intervals are marked by the error bars.

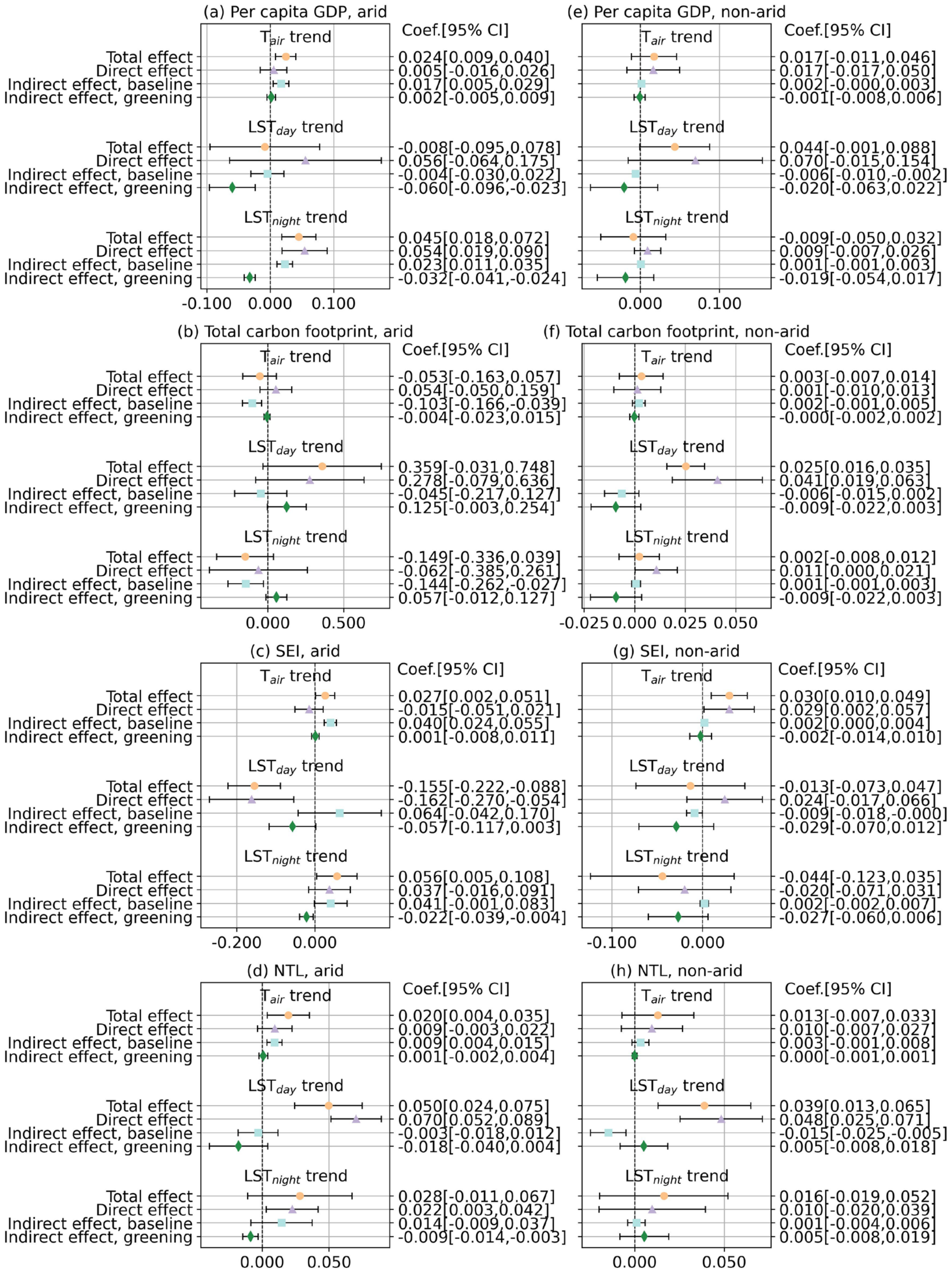

Figure S4 Stratified analysis by (a-d) arid and (e-h) non-arid cities, showing total, direct, and indirect effects of per capita GDP, total carbon footprint, social environment index (SEI), and nighttime light intensity (NTL) on temperature trends. More developed cities are those within the top 50% of the corresponding socioeconomic variable, whereas less developed cities are the rest. The bars represent changes in temperature trend (°C/decade) for a one-standard-deviation increase in the corresponding covariate, when holding other covariates constant. Models estimating total effect adjust for climate zone, coastal adjacency, land elevation, population density, total urban area, and country-fixed effects. Models estimating direct and indirect effects additionally control for temperature, greenness, and greening. 95% confidence intervals are marked by the error bars.

References

Bilal, U., Hessel, P., Perez-Ferrer, C., Michael, Y. L., Alfaro, T., Tenorio-Mucha, J., Friche, A. A. L., Pina, M. F., Vives, A., Quick, H., Alazraqui, M., Rodriguez, D. A., Miranda, J. J., & Diez-Roux, A. V. (2021). Life expectancy and mortality in 363 cities of Latin America. *Nature Medicine*, *27*(3), 463–470. https://doi.org/10.1038/s41591-020-01214-4

Copernicus Climate Change Service (C3S). (2019). *ERA5-Land hourly data from 2001 to present* [Data set]. ECMWF. https://doi.org/10.24381/CDS.E2161BAC

Esch, T., Bachofer, F., Heldens, W., Hirner, A., Marconcini, M., Palacios-Lopez, D., Roth, A., Üreyen, S., Zeidler, J., Dech, S., & Gorelick, N. (2018). Where We Live—A Summary of the Achievements and Planned Evolution of the Global Urban Footprint. *Remote Sensing*, *10*(6), Article 6. https://doi.org/10.3390/rs10060895

Farr, T. G., Rosen, P. A., Caro, E., Crippen, R., Duren, R., Hensley, S., Kobrick, M., Paller, M., Rodriguez, E., Roth, L., Seal, D., Shaffer, S., Shimada, J., Umland, J., Werner, M., Oskin, M., Burbank, D., & Alsdorf, D. (2007). The Shuttle Radar Topography Mission. *Reviews of Geophysics*, *45*(2). https://doi.org/10.1029/2005RG000183

Hu, L., & Bentler, P. M. (1999). Cutoff criteria for fit indexes in covariance structure analysis: Conventional criteria versus new alternatives. *Structural Equation Modeling: A Multidisciplinary Journal*, *6*(1), 1–55. https://doi.org/10.1080/10705519909540118

Kummu, M., Taka, M., & Guillaume, J. H. A. (2018). Gridded global datasets for Gross Domestic Product and Human Development Index over 1990–2015. *Scientific Data*, *5*(1), Article 1. https://doi.org/10.1038/sdata.2018.4

Moran, D., Kanemoto, K., Jiborn, M., Wood, R., Többen, J., & Seto, K. C. (2018). Carbon footprints of 13,000 cities. *Environmental Research Letters*, *13*(6), 064041. https://doi.org/10.1088/1748-9326/aac72a

NOAA National Centers for Environmental Information. (2017). *Shoreline/Coastline Databases*. U.S. Department of Commerce. https://www.ngdc.noaa.gov/mgg/shorelines/

Quistberg, D. A., Diez Roux, A. V., Bilal, U., Moore, K., Ortigoza, A., Rodriguez, D. A., Sarmiento, O. L., Frenz, P., Friche, A. A., Caiaffa, W. T., Vives, A., Miranda, J. J., & SALURBAL Group. (2018). Building a Data Platform for Cross-Country Urban Health Studies: The SALURBAL Study. *Journal of Urban Health: Bulletin of the New York Academy of Medicine*, *96*. https://doi.org/10.1007/s11524-018-00326-0

Shi, D., Lee, T., & Maydeu-Olivares, A. (2019). Understanding the Model Size Effect on SEM Fit Indices. *Educational and Psychological Measurement*, *79*(2), 310–334. https://doi.org/10.1177/0013164418783530

Wessel, P., & Smith, W. H. F. (1996). A global, self-consistent, hierarchical, high-resolution shoreline database. *Journal of Geophysical Research: Solid Earth*, *101*(B4), 8741–8743. https://doi.org/10.1029/96JB00104
